# Your brain doesn’t look a day past 70! Cross-sectional associations with brain-predicted age in the cognitively-intact oldest-old

**DOI:** 10.1007/s11357-025-02027-4

**Published:** 2025-12-11

**Authors:** Mark K. Britton, Hannah Hoogerwoerd, Joshua Juhasz, Keyanni Joy Johnson, Paul D. Stewart, Pradyumna K. Bharadwaj, Stacy S. Merritt, Cortney J. Jessup, Clinton B. Wright, G. Alex Hishaw, David A. Raichlen, Victor A. Del Bene, Virginia G. Wadley, Theodore P. Trouard, Noam Alperin, Bonnie E. Levin, Tatjana Rundek, Kristina M. Visscher, Gene E. Alexander, Ronald A. Cohen, Eric C. Porges, Joseph M. Gullett

**Affiliations:** 1 Department of Epidemiology, University of Florida, Gainesville, FL 32610 USA; 2 Center for Cognitive Aging and Memory, Evelyn F. and William L. McKnight Brain Institute, University of Florida, Gainesville, FL 32610 USA; 3 Department of Clinical and Health Psychology, University of Florida, Gainesville, FL 32610 USA; 4 Department of Neurobiology, University of Alabama at Birmingham, Birmingham, Alabama, 35233 USA; 5 Evelyn F. McKnight Brain Institute, University of Alabama at Birmingham, Birmingham, Alabama 35233 USA; 6 Department of Psychology, University of Arizona, Tucson, AZ 85719 USA; 7 Evelyn F. McKnight Brain Institute, University of Arizona, Tucson, AZ 85719 USA; 8 Department of Neurology, University of Miami, Miami, FL 33136 USA; 9 Evelyn F. McKnight Brain Institute, University of Miami, Miami, FL 33136 USA; 10 National Institute of Neurological Disorders and Stroke, National Institutes of Health, Bethesda, MD 20824 USA; 11 Department of Neurology, University of Arizona, Tucson, AZ 85719 USA; 12 Department of Biological Sciences, University of Southern California, Los Angeles, CA 90089 USA; 13 Department of Neurology, University of Alabama at Birmingham, Birmingham, Alabama 35233 USA; 14 Heersink School of Medicine, University of Alabama at Birmingham, Birmingham, Alabama 35233 USA; 15 Department of Biomedical Engineering, University of Arizona, Tucson, AZ 85719 USA; 16 Department of Radiology, University of Miami, Miami, FL 33136 USA; 17 Department of Radiology and Biomedical Imaging, Yale University, New Haven, CT USA

**Keywords:** Magnetic resonance imaging, Supervised machine learning, Longevity, Cognitive aging, Epidemiologic factors, Selection bias

## Abstract

The cognitively-intact oldest-old (85 +) may be the most-resilient members of their birth cohort; due to survivorship effects (e.g., depletion of susceptibles), risk factors associated with brain aging biomarkers in younger samples may not generalize to the cognitively-intact oldest-old. We evaluated associations between established aging-related risk factors and brain-predicted age difference (brainPAD) in a cross-sectional cognitively-intact oldest-old sample. Additionally, we evaluated brainPAD-cognition associations to characterize brain maintenance vs. cognitive reserve in our sample. Oldest-old adults (N = 206; 85–99 years; Montreal Cognitive Assessment > 22 or neurologist evaluation) underwent T1-weighted MRI; brainPAD was generated with brainageR, such that more-positive brainPAD reflected more-advanced brain aging. Sex, education, alcohol and smoking history, exercise history, BMI, cardiovascular and metabolic disease history, and anticholinergic medication burden were self-reported. Global cognitive z-score and coefficient of variation were derived from the UDS 3.0 cognitive battery; crystallized-fluid discrepancy was derived from the NIH Toolbox Cognitive Battery. Mean brainPAD was -7.99 (SD: 5.37; range: -24.50, 6.03). Women showed more-delayed brain aging than men (B = -2.9, 95% CI = -4.6, -1.1, p = 0.002). No other exposures were significantly associated with brainPAD. BrainPAD was not associated with any cognitive variable. These findings suggest that cognitively-intact oldest-old adults may be atypically-resistant to risk factors associated with aging in younger samples, consistent with survivorship effects in aging. Furthermore, brainPAD may have limited explanatory value for cognitive performance in cognitively-intact oldest-old adults, potentially due to high cognitive reserve. Overall, our findings highlight the impact of survivorship effects on brain aging research.

**Graphical Abstract:**

**Figure:**
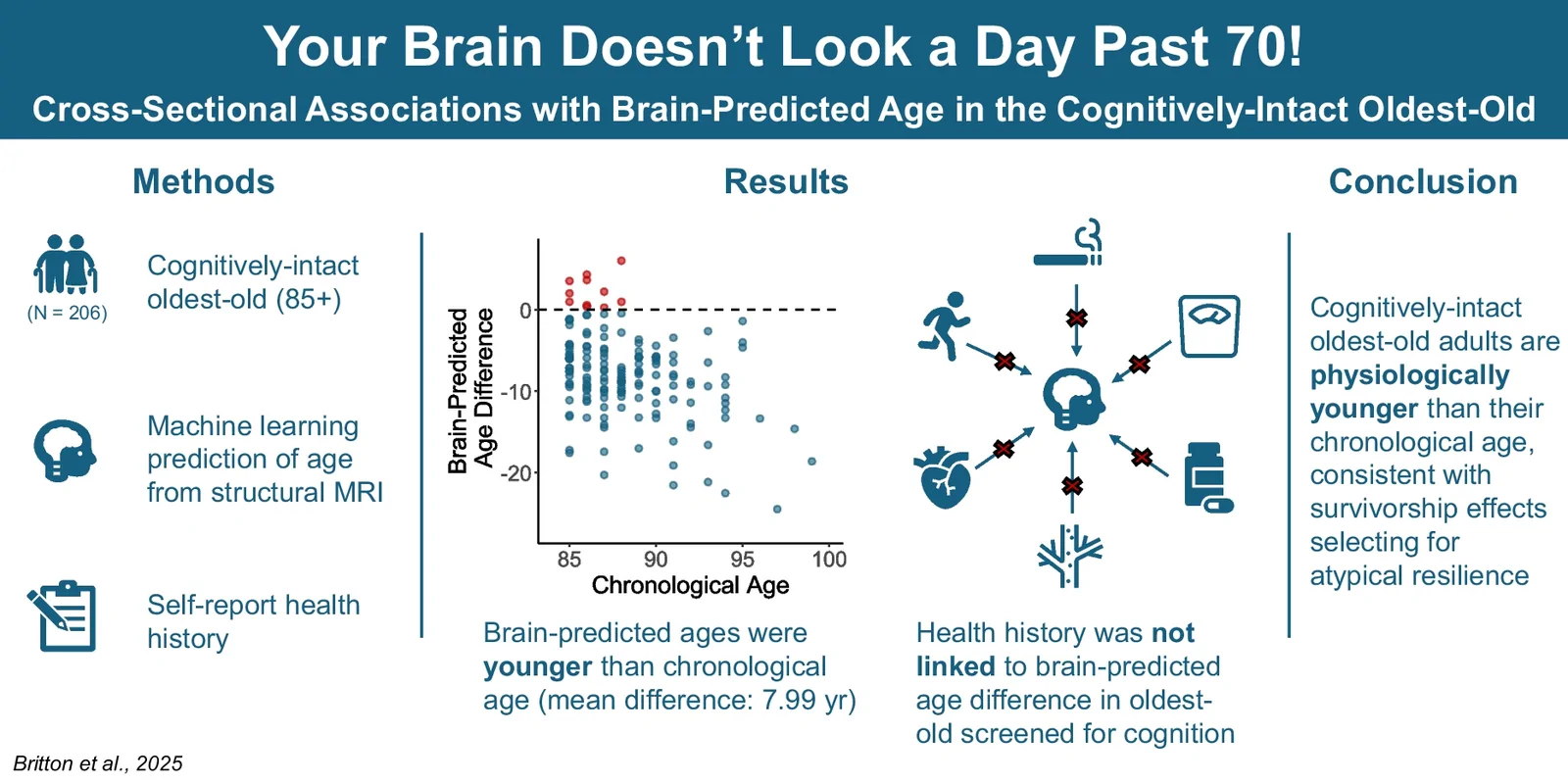

**Supplementary Information:**

The online version contains supplementary material available at https://doi.org/10.1007/s11357-025-02027-4.

## Introduction

Oldest-old adults (≥ 85 years) are, by definition, survivors among their birth cohort. An estimated 23% of men and 38% of women born in the United States in 1930 reached the age of 85 [1]. Of this minority, only a subset maintain largely-intact cognitive function into oldest-old age: the 2022 National Health Interview Survey reported that 13.1% of noninstitutionalized US adults 85 years and older had ever been diagnosed with dementia [2], while estimates of Mild Cognitive Impairment (MCI) burden in noninstitutionalized adults 80 years and older range from 16–27% [3]. However, some oldest-old adults do maintain intact cognitive performance, whether due to physiological resistance to aging (brain maintenance) or strong compensatory abilities (i.e., cognitive reserve) [4, 5]. Consequently, a growing body of research has sought to identify protective factors associated with brain function in cognitively-intact oldest-old adults, with the goal of identifying potential interventions to prolong cognitive function in aging [6, 7].

Brain-predicted age is an emerging biomarker of overall physiological brain aging [8, 9]. Brain-predicted age refers to a chronological age value predicted from a structural brain image (e.g., a T1-weighted MRI) using supervised machine learning regression. Brain-predicted age difference (brainPAD), or the difference between chronological age and brain-predicted age, has been interpreted as a biomarker of advanced vs. delayed brain aging in middle-aged and younger old (i.e., 65–84) adults [8]. brainPAD is associated with midlife exposures linked to physiological aging, such as physical activity and fitness [10–12], cardiovascular [13–16] and metabolic health [16–22], smoking [12, 23, 24], and greater alcohol use [24–27]. Furthermore, more-positive brainPAD (i.e., advanced brain age) has been linked to worse cognition in older adults [28–32] and outperforms total brain volume and Mini-Mental State Examination score as a predictor of dementia hazard in at-risk older adults [33, 34]. In sum, brainPAD is a potentially informative biomarker of physiological brain aging and its relationship to cognition in oldest-old adults.

Despite brainPAD’s potential utility in this population, relatively few studies of brainPAD have included oldest-old adults [30, 32, 35–37] and, to our knowledge, none have reported data including only oldest-old adults. However, associations reported in younger old adults may not generalize to the oldest-old in general or to the cognitively-intact oldest-old. Although structural brain aging in oldest-old adults is generally understudied [38], age effects on brain macrostructure have been suggested to decrease nonlinearly in oldest-old age: for instance, in cross-sectional data, oldest-old adults show weaker age effects on white matter volume [39] and sulcal widening [40] relative to younger old adults. We have previously reported similar attenuation of age effects on neurotransmitter levels in the oldest-old [41], consistent with relative brain preservation in this population.

The apparent attenuation of age effects on brain structure and function in cross-sectional samples of the oldest-old may be partly explained by survivorship effects. Individuals who have survived to oldest-old age are likely atypically healthy or resilient [42–44]; this is especially true for the cognitively-intact oldest-old. Cognitively-intact oldest-old individuals may be resistant to age-related brain changes (brain maintenance) or more able to compensate for those changes (cognitive reserve), potentially buffering the effects of aging or lifestyle exposures [38, 44, 45]. Conversely, the individuals most susceptible to age-related or lifestyle-related brain changes are likely to have been eliminated from the sampled population due to earlier mortality or earlier onset of cognitive impairment (i.e., depletion of susceptibles) [42]. Attrition of less-healthy individuals may therefore attenuate associations between risk factors and brain structure and function with increasing age [46]; for instance, associations between diabetes and dementia [47], as well as APOE4 and dementia [48, 49], may not generalize from younger old samples to individuals who have survived to oldest-old age without incident cognitive impairment. It is currently unknown to what extent exposure-brainPAD associations previously-reported in other age strata generalize to the cognitively-intact oldest-old.

The current secondary analysis aims to fill this literature gap by assessing exposure-brainPAD associations in a cross-sectional sample of cognitively-intact oldest-old adults. Lifetime substance use, lifetime history of cardiovascular and metabolic disorders, lifetime physical activity, Body Mass Index (BMI), and educational history were selected for analysis due to reported associations with brainPAD in younger old samples; anticholinergic medication burden was selected due to reported associations between anticholinergic use and brain aging [50] as well as cognitive decline and dementia studied both cross-sectionally [51] and longitudinally [52]. We hypothesized that greater use of cigarettes and alcohol, greater lifetime history of cardiovascular and metabolic disorders, lower lifetime physical activity, greater BMI, lower educational attainment, and greater anticholinergic burden would be associated with an older-appearing brain. As a second aim, to differentiate brain maintenance from compensatory ability in our sample, we examined associations between brainPAD and global cognitive performance, cognitive coefficient of variation (a measure of intra-individual variability, which has been linked to cognitive aging and dementia) [53, 54], and fluid-crystallized cognition discrepancy (an index of cognitive decline) [55]. We hypothesized that a younger-appearing brain would be linked to better overall cognition, more consistent cognitive performance, and smaller fluid-crystallized discrepancy, consistent with successful brain maintenance.

## Methods

### Participants

Oldest-old adults (N = 206) were recruited at four study sites as part of a larger parent study of brain and cognitive health in the oldest-old (McKnight Brain Aging Registry). All participants were 85 years of age or older and were cognitively-intact as determined by either scores of 28 or more on the Telephone Interview for Cognitive Status – Modified (TICS-M) and 22 or more on the Montreal Cognitive Assessment (MoCA) or a neurologist’s evaluation. Study exclusion criteria were the following: major physical disability; medical conditions expected to limit life expectancy or interfere with study participation (e.g., unstable cancer or uncontrolled severe Major Depressive Disorder); current DSM-5 substance use disorder (SUD); inability to independently perform instrumental activities of daily living (IADLs) or basic activities of daily living (ADLs); less than sixth-grade reading level; or any condition that would interfere with study procedures (e.g., hearing or vision loss, MRI contraindications). All participants provided written informed consent. Study procedures were approved by Institutional Review Boards at the University of Florida (#201300162), University of Miami (#20151783), University of Arizona (#1601318818), and University of Alabama at Birmingham (#X160113004) and were conducted in accordance with the Declaration of Helsinki.

### Demographic and clinical variables

Exposure variables of interest were age, assigned sex, years of education, race, Hispanic/Latino ethnicity, estimated lifetime cigarette pack-years (never-smoker/< median/≥ median), lifetime alcohol abuse (yes/no), current standard drinks per week (none/< 7/≥ 7), BMI, cumulative lifetime physical activity quartile, current use of any anticholinergic medication, and lifetime diagnosis (yes/no) of the following medical conditions: hypertension; high cholesterol; diabetes mellitus; myocardial infarction; or cardiac arrest. All variables were derived from self-report. Data cleaning approaches are reported in detail in Supplementary Methods.

### Neuropsychological testing

Participants completed the Uniform Data Set (UDS 3.0) neurocognitive testing battery [56]. z-scores were generated for each measure using the regression-based UDS 3.0 norms corrected for sex, age, and years of education [56]. The mean z-score across all measures for each participant was used as an index of global cognitive performance. A full list of measures is reported in Table 1.

**Table 1:** Measures taken from the UDS 3.0 neuropsychological battery

Global Cognitive z-Score	Coefficient of Variation	Domain
Craft Story 21 immediate recall, verbatim	Craft Story 21 immediate recall, verbatim	Memory
Craft Story 21 immediate recall, paraphrase	-	Memory
Benson complex figure copy, total score	Benson complex figure copy, total score	Visuospatial
Number span test forward, total correct	Number span test forward, total correct	Attention
Number span test forward, longest span	-	Attention
Number span test backward, total correct	Number span test backward, total correct	Attention
Number span test backward, longest span	-	Attention
Animals list generation, total in 60 s	Animals list generation, total in 60 s	Language (category fluency)
Vegetables list generation, total in 60 s	Vegetables list generation, total in 60 s	Language (category fluency)
Trail-Making Test, Part A	Trail-Making Test, Part A	Processing speed
Trail-Making Test, Part B	Trail-Making Test, Part B	Executive function
Craft Story 21 delayed recall, verbatim	Craft Story 21 delayed recall, verbatim	Memory
Craft Story 21 delayed recall, paraphrase	-	Memory
Benson complex figure recall, total score	Benson complex figure recall, total score	Visuospatial/Memory
MINT total score	MINT total score	Language (naming)
Phonemic test, total F-words and L-words	Phonemic test, total F-words and L-words	Language (verbal fluency)

Coefficient of variation (CoV) for a subset of the measures included in the UDS 3.0 (Table 1) was used as an index of cognitive dispersion. Greater cognitive dispersion has been argued to reflect failure of top-down executive control mechanisms [57] and may have prognostic value for future cognitive decline [58]. The CoV was calculated using demographically-adjusted scores, following the approach used for the global mean z-score. For primary analyses, the CoV was then further adjusted using published norms for this composite in the UDS 3.0 battery [59]; these norms are corrected for age, years of education, sex, and race/ethnicity (non-Hispanic White/other) and are available from 50–101 years of age [59]. To maintain consistency with the norming approach used for the global mean z-score, supplementary analyses were performed without applying further demographic adjustment to the CoV composite.

Participants additionally completed the NIH Toolbox Cognition Battery. Crystallized-fluid cognition discrepancy was used as a proxy for cognitive decline [55]. NIH Toolbox Fluid Cognition Composite was subtracted from NIH Toolbox Crystallized Cognition Composite to calculate crystallized-fluid discrepancy [60], a metric of decline from estimated premorbid intellect. Because age-corrected NIH Toolbox Cognition Battery norms are not available for individuals older than 85 [61], uncorrected Standard Scores were converted to uncorrected z-scores (to maintain consistency with other cognitive summary scores) and used for analyses of NIH Toolbox data.

### Structural image acquisition and processing

T1-weighted MP-RAGE images were acquired on a Siemens Prisma or Skyra 3 T scanner, using a 64-channel head coil, according to a standard protocol across sites (TE: 3.37 ms; TR: 2530 ms; flip angle = 7°; FoV = 240 × 256 x 176 mm; voxel size: 1.0 × 1.0x1.0mm^3^; scan duration: 6 min and 3 s). Images were quality-checked visually by a trained rater.

Images were then submitted to the automated brainageR 2.1 pipeline [62]. Images were segmented into gray matter, white matter, and CSF, normalized in SPM12 [63], and visually quality-checked again; segmented GM, WM, and CSF images were then vectorized in R 4.2.2, masked to the brainageR template, and submitted to brainageR’s PCA rotation matrix. The 435 principal components produced by rotation were then entered as predictors into a Gaussian Process regression model using kernlab. Model output was brain-predicted age. brainPAD was calculated as brain-predicted age – chronological age. No deviations were made from the default brainageR pipeline.

To evaluate the replicability of our findings across algorithms, additional brain-predicted age estimates were generated using two additional publicly-available and widely-used age prediction algorithms, xgBoost [64] and pyment [65]; these algorithms were selected due to high sensitivity to cognitive impairment in mixed younger-old and oldest-old samples [37] and accurate prediction of age progression in longitudinal studies [66], respectively. Full details of these pipelines are provided in Supplementary Methods. brainPAD_xgBoost_ and brainPAD_pyment_ were calculated as brain-predicted age – chronological age, following the approach used to calculate brainPAD.

### Statistical analysis

All analyses were conducted in R 4.3.1 [67]. Distribution of clinical and demographic variables of interest was reported descriptively by study site. Sex differences in chronological age were assessed with a t-test. Pearson correlation between brainPAD and chronological age was reported for all three machine learning algorithms (brainageR, xgBoost, and pyment). To address bias created by missing data or poor-quality MR images, covariate imbalance between complete and incomplete cases was assessed descriptively using standardized mean differences (continuous variables) and raw differences in proportion (categorical variables) in the cobalt R package [68]; a threshold of 0.10 was used as a cutoff for imbalance. Due to the high proportion of incomplete cases (42%), incomplete cases were imputed under fully-conditional specification using mice 3.17.0 [69]. Full details on the imputation procedure are reported in Supplementary Methods. Lifetime exercise and pack-years were categorized separately for each imputation.

To address our first aim, brainPAD was entered as the outcome of a linear model. A random site-specific intercept was initially fit in lmerTest [70] to address site effects; however, the random intercepts were dropped from analyses due to explained variance approaching zero (i.e., no substantial variance explained by site-specific intercepts) in both imputed and unimputed data. Fixed predictor terms were age, sex, years of education, cigarette pack-year history (nonsmoker/< median/≥ median), drinks per week (none/< 7/≥ 7), exercise quartile, history of hypercholesterolemia diagnosis, history of hypertension diagnosis, BMI, and anticholinergic medication use. Diabetes, myocardial infarction, cardiac arrest, Hispanic ethnicity, non-White race, and lifetime alcohol abuse were dropped as predictors due to low frequency in the sample (≤ 10% each in unimputed data). All continuous variables were entered as linear terms, as inspection of scatterplots and model residuals did not suggest quadratic or cubic relationships. Parameters of interest were estimated separately for individual imputed datasets and then pooled using Rubin’s rules. Three supplementary analyses were conducted: first, the model was repeated on unimputed data only, to assess the impact of multiple imputation on observed results; second, a sex-education interaction term was added to the original model to assess sex differences in the effects of education; and third, the original model was repeated with brainPAD_xgBoost_ and brainPAD_pyment_ entered as dependent variables.

To address our second aim, brainPAD-cognition associations were evaluated with linear mixed effects models fit in lmerTest [70]. Separate models were fit with each cognitive summary score (global cognitive z-score, CoV, and fluid-crystallized discrepancy) as dependent variables. BrainPAD and age were entered as fixed predictor terms in all models. Again, inspection of scatterplots and model residuals did not indicate broadly nonlinear associations. Due to the use of uncorrected scores for NIH Toolbox Fluid-Crystallized Discrepancy, sex and years of education were included as covariates in this model only. Separate random intercepts were fit for each study site. Again, parameters of interest were estimated separately for individual imputed datasets and then pooled using Rubin’s rules. Supplementary analyses were conducted using a listwise deletion approach to assess the impact of imputation on results. Additional supplementary analyses were conducted using an alternate demographic correction approach for CoV (as reported in Sect. 2.3).

## Results

###   Clinical and demographic variables

Clinical and demographic variables are reported by site in Table 2 (unimputed data). Participants were predominantly non-Hispanic White, with a mean of 16.11 (SD: 2.98) years of education. Forty-five percent of participants were male. Among men, the mean chronological age was 88.32 years (SD: 3.34); among women, the mean chronological age was 88.54 years (SD: 2.96; t(187.70) = −0.49, p = 0.63, effect size *d* = −0.07). Among participants with current alcohol use data prior to imputation (N = 200), 2% (N = 4) reported exceeding NIAAA drinks/week cutoffs for heavy drinking (> 7 for females and > 14 for males) [71].

**Table 2:** Demographic characteristics and self-reported medical history of MBAR participants, unimputed data (N = 206)

Characteristic	Overall *N* = 206^a^	Site 1 *N* = 58^a^	Site 2 *N* = 45^a^	Site 3 *N* = 49^a^	Site 4 *N* = 54^a^
Age	88.44 (3.13)	88.55 (3.45)	88.49 (2.88)	89.12 (3.43)	87.65 (2.53)
Female	112 (54%)	29 (50%)	22 (49%)	29 (59%)	32 (59%)
Race					
Asian	2 (1.0%)	0 (0%)	0 (0%)	1 (2.0%)	1 (1.9%)
Black/African American	6 (2.9%)	4 (6.9%)	0 (0%)	0 (0%)	2 (3.7%)
Other	1 (0.5%)	0 (0%)	0 (0%)	1 (2.0%)	0 (0%)
White	197 (96%)	54 (93%)	45 (100%)	47 (96%)	51 (94%)
Hispanic/Latino	6 (2.9%)	0 (0%)	2 (4.4%)	0 (0%)	4 (7.4%)
Years of Education	16.11 (2.98)	15.90 (2.67)	16.09 (2.87)	16.29 (3.35)	16.19 (3.11)
MoCA Score	24.77 (2.49)	24.47 (2.11)	24.36 (2.53)	24.94 (2.82)	25.28 (2.47)
Missing	1	0	0	0	1
Smoking Pack-Years					
Nonsmoker	94 (49%)	26 (46%)	18 (41%)	21 (49%)	29 (62%)
High	47 (25%)	15 (26%)	13 (30%)	11 (26%)	8 (17%)
Low	50 (26%)	16 (28%)	13 (30%)	11 (26%)	10 (21%)
Missing	15	1	1	6	7
Current Alcohol Use					
None	72 (36%)	30 (53%)	9 (20%)	20 (41%)	13 (27%)
< 7 Drinks/Week	80 (40%)	17 (30%)	24 (53%)	19 (39%)	20 (41%)
≥ 7 Drinks/Week	48 (24%)	10 (18%)	12 (27%)	10 (20%)	16 (33%)
Missing	6	1	0	0	5
Lifetime Alcohol Abuse	2 (1.0%)	0 (0%)	0 (0%)	2 (4.1%)	0 (0%)
Missing	4	0	0	0	4
Lifetime Diabetes Diagnosis^b^	21 (10%)	9 (16%)	5 (11%)	5 (10%)	2 (3.7%)
Lifetime Hypertension Diagnosis	120 (59%)	36 (62%)	25 (56%)	32 (65%)	27 (54%)
Missing	4	0	0	0	4
Lifetime High Cholesterol Diagnosis	99 (48%)	30 (52%)	24 (53%)	19 (39%)	26 (48%)
Lifetime Myocardial Infarction	4 (2.0%)	3 (5.2%)	0 (0%)	1 (2.0%)	0 (0%)
Missing	6	0	0	0	6
Lifetime Cardiac Arrest	3 (1.5%)	2 (3.4%)	0 (0%)	1 (2.0%)	0 (0%)
Missing	6	0	0	0	6
Body Mass Index	25.24 (3.70)	25.62 (3.45)	25.48 (3.94)	24.97 (4.09)	24.86 (3.41)
Missing	3	0	0	0	3
Anticholinergic Cognitive Burden Scale	0.63 (1.08)	0.98 (1.48)	0.38 (0.65)	0.53 (1.04)	0.53 (0.74)
Missing	8	1	0	2	5

^a^Mean (SD); n (%)

^b^Type 2 (N = 19) or unspecified (N = 2)

### Missingness

Prior to imputation, 157 (76%) participants had interpretable brainPAD data and 113 (55%) had complete data for all variables of interest. Missingness is reported in full in Supplementary Table 2. Missingness was associated with study site, lower education, lower MoCA score, greater BMI, heavier smoking, higher lifetime exercise quartile, greater anticholinergic burden, lower brainageR-derived brain-predicted age, and higher xgBoost- and pyment-derived brain-predicted age (Supplementary Table 3).

### BrainPAD

**Fig. 1:**
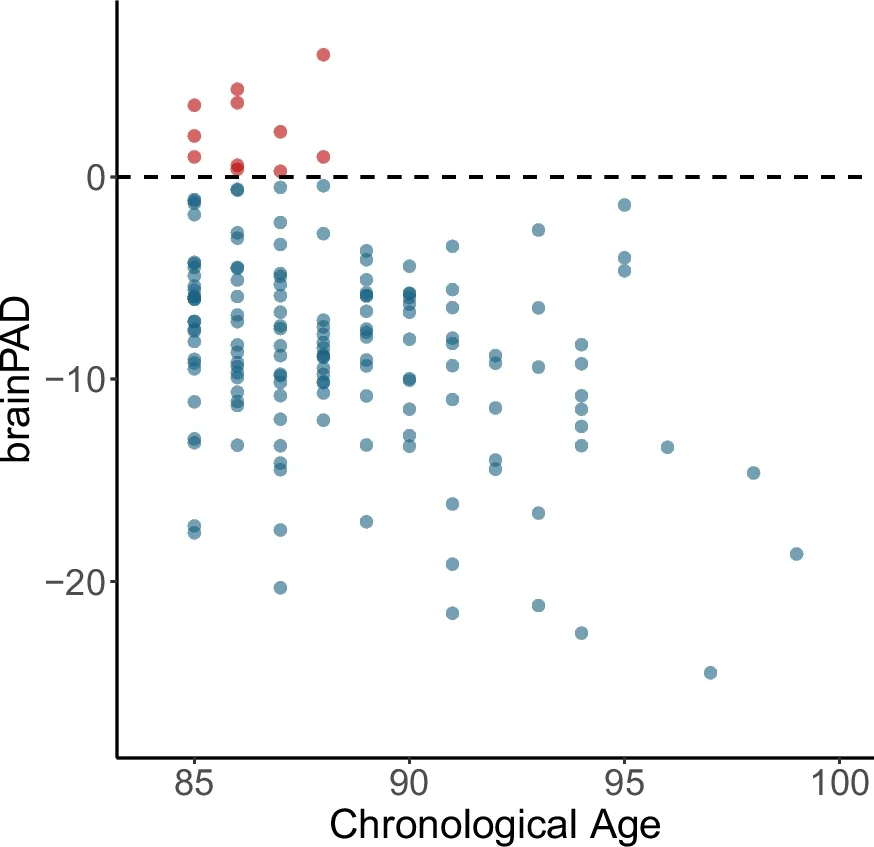
Visualization of brainPAD and chronological age in cognitively-intact oldest-old adults, unimputed data (N = 157). Mean brainPAD was −7.99 (SD: 5.37, range: −24.50, 6.03), representing a roughly 8-year delay in brain aging

**Fig. 2:**
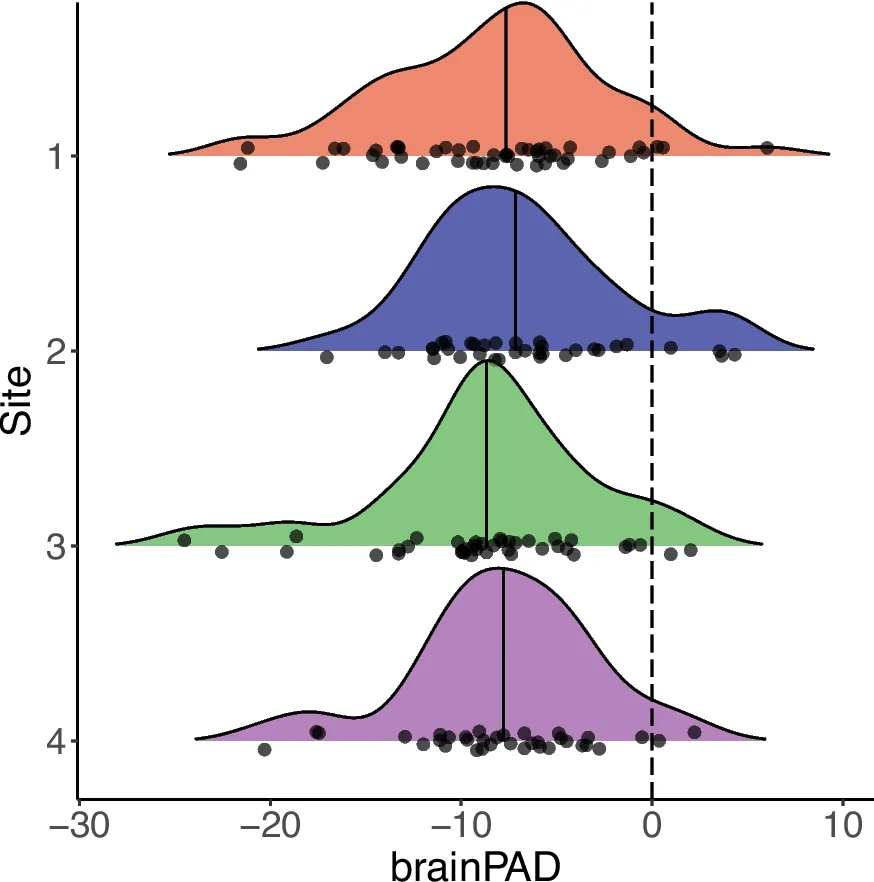
Distribution of brainPAD by study site in cognitively-intact oldest-old adults (85 +), unimputed data (N = 157)

In unimputed data, the Pearson correlation between brainageR-derived predicted age and chronological age was 0.21 (95% CI = 0.05, 0.35, p = 0.009). The mean brainPAD was −7.99 (SD: 5.37; range: −24.50, 6.03; Fig. 1). The distribution of brainPAD by site is plotted in Fig. 2.

Supplementary analyses revealed that the Pearson correlation between xgBoost-derived brain-predicted age and chronological age was 0.05 in unimputed data (95% CI = −0.11, 0.21, p = 0.54); the mean brainPAD_xgBoost_ was −20.57 (SD: 4.18; range: −35.00, −11.75). Likewise, the Pearson correlation between pyment-derived brain-predicted age and chronological age was 0.21 (95% CI = 0.05, 0.35, p = 0.01); the mean brainPAD_pyment_ was −14.31 (SD: 4.92; range: −37.48, −1.79). Distributions of supplementary brainPAD estimates are visualized in Supplementary Fig. 1.

### Lifetime exposure history and brainPAD

**Fig. 3:**
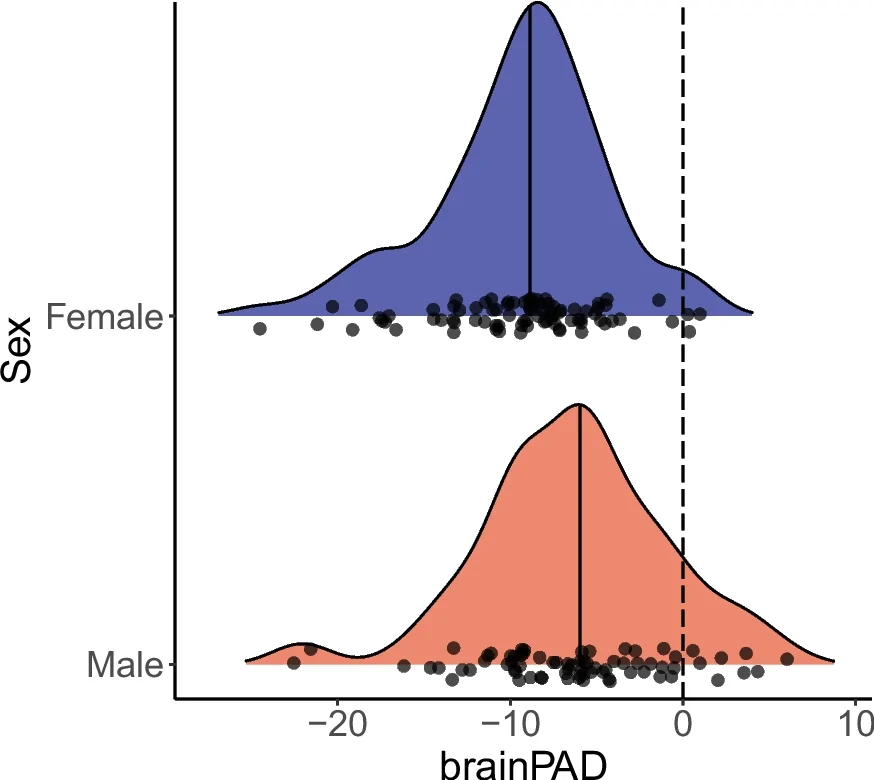
Among cognitively-intact oldest-old adults (85 +), brainPAD was significantly more negative in females, unimputed data (N = 157)

Results of linear models on imputed data are reported in Table 3. Female sex was significantly associated with more-negative brainPAD (B = −2.9, 95% CI = −4.64, −1.10, p = 0.002; Fig. 3); that is, female participants had relatively younger-appearing brains (mean: −9.42; SD: 4.90) compared to males (mean: −6.34; SD: 5.43). Age was inversely associated with brainPAD (B = −0.66, 95% CI = −0.93, −0.40, p < 0.001), such that older participants showed comparatively-delayed brain aging. No other predictor variables were significantly associated with brainPAD (Table 3). Results were substantively similar in complete-case analyses (Supplementary Table 3).

**Table 3:** Results of linear model predicting brainPAD from participant characteristics (N = 206), imputed data. Site-specific random intercept terms were dropped due to low variance explained

Characteristic	B	95% CI^a^	p
Age	−0.66	−0.93, −0.40	< 0.001
Female	−2.9	−4.6, −1.1	0.002
Smoking Pack-Years			
Nonsmoker	—	—	
High	−0.7	−2.8, 1.4	0.5
Low	−1.0	−3.0, 1.0	0.3
Anticholinergic Medication Use	−0.24	−2.0, 1.5	0.8
Cumulative Lifetime Exercise Quartile			
Q1	—	—	
Q2	−0.61	−2.7, 1.4	0.5
Q3	−0.53	−2.9, 1.8	0.7
Q4	−0.15	−2.5, 2.1	0.9
Current Drinks/Week			
< 7	—	—	
≥ 7	−0.38	−2.4, 1.7	0.7
None	−1.3	−3.1, 0.52	0.2
Lifetime Hypertension Diagnosis	0.31	−1.4, 2.0	0.7
Lifetime High Cholesterol Diagnosis	−0.53	−2.2, 1.2	0.5
Years of Education	0.02	−0.25, 0.29	0.9
BMI	0.05	−0.18, 0.29	0.7

^a^CI = Confidence Interval

In supplementary models, sex was not significantly associated with brainPAD_xgBoost_ (B = −0.62, 95% CI = −1.73, 0.48, p = 0.26); in unimputed data, mean brainPAD_xgBoost_ was −19.70 (SD: 4.01) for males and −21.34 (SD: 4.19) for females. Results were otherwise substantively similar to findings from main models (Supplementary Table 4). Likewise, sex was not associated with brainPAD_pyment_ (B = −0.67, 95% CI = −2.41, 1.06, p = 0.44); in unimputed data, mean brainPAD_pyment_ was −13.75 (SD: 5.30) for males and −14.81 (SD: 4.53) for females. Other associations from this model were broadly similar to results from the main model (Supplementary Table 4).

In supplementary models including a sex-by-education interaction term, the precision of coefficient estimates for sex was attenuated; however, other results were broadly similar (Supplementary Table 5).

### BrainPAD and cognitive performance

**Fig. 4:**
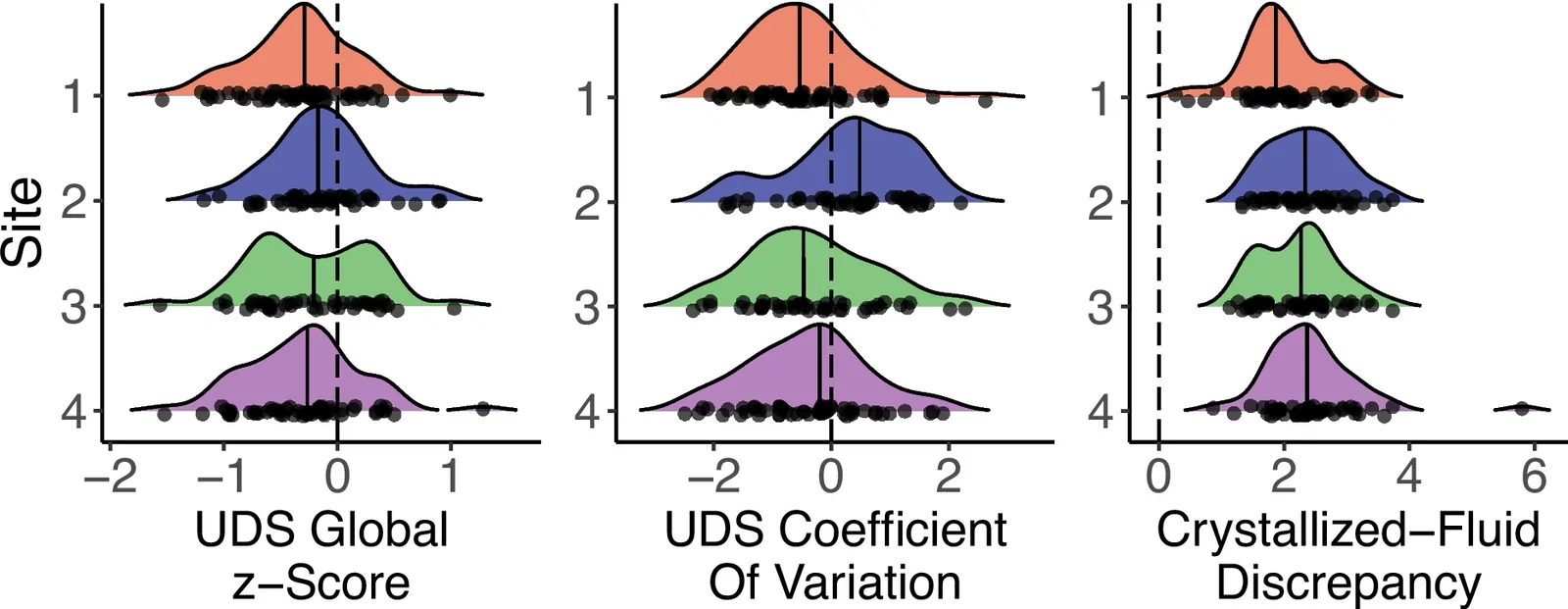
Distribution of UDS 3.0 and NIH Toolbox cognitive summary variables by study site, unimputed data. Variables are reported as z-scores

The distribution of UDS 3.0 global z-score, UDS 3.0 CoV, and NIH Toolbox Crystallized-Fluid Discrepancy Score by site is visualized in Fig. 4. In unimputed data, the mean UDS 3.0 global z-score was −0.26 (SD: 0.49); the mean UDS 3.0 CoV was −0.26 (SD: 1.04); and the mean NIH Toolbox Crystallized-Fluid Discrepancy z-score was 2.23 (SD: 0.69).

Results of linear mixed effects models on imputed data are reported in Table 4. There was no statistically-significant association between brainPAD and UDS 3.0 global z-score (B = −0.005, 95% CI = −0.02, 0.01, p = 0.55) or UDS 3.0 CoV (B = 0.01, 95% CI = −0.02, 0.04, p = 0.62) in models adjusted for age. There was no statistically-significant association between brainPAD and NIH Toolbox Crystallized-Fluid Discrepancy (B = 0.002, 95% CI = −0.02, 0.02, p = 0.84) in models adjusted for age, sex, and years of education. Results were substantively similar in complete-case analyses (Supplementary Table 6) and in analyses using an alternate demographic correction approach for CoV (Supplementary Table 7).

**Table 4:** Fixed effects from linear mixed effects models of brainPAD and cognitive performance (N = 206), imputed data

	UDS 3.0 Global z-score^a^			UDS 3.0 Coefficient of Variation^b^			NIH Toolbox Crystallized-Fluid Discrepancy^c^		
Characteristic	B	95% CI^d^	p	B	95% CI^d^	p	B	95% CI^d^	p
Age	−0.006	−0.03, 0.02	0.62	0.01	−0.04, 0.06	0.82	0.03	−0.006, 0.65	0.10
brainPAD	−0.005	−0.02, 0.01	0.55	0.01	−0.02, 0.04	0.64	0.002	−0.02, 0.02	0.84
Years of Education	—	—	—	—	—	—	0.05	0.02, 0.08	0.004
Female	—	—	—	—	—	—	0.08	−0.13, 0.29	0.46

^a^Adjusted for age, years of education, and sex using standard UDS 3.0 norms

^b^Adjusted for age, years of education, sex, and race/ethnicity using published norms for this composite

^c^Uncorrected scores were used due to unavailability of age-corrected NIH Toolbox norms for individuals over 85

^d^CI = Confidence Interval

## Discussion

Although brainPAD is a promising biomarker of age-related differences in brain structure, it has not been extensively examined in oldest-old adults to date. Cross-sectional associations between exposure history, biomarkers of aging, and cognitive performance may not generalize from younger old to oldest-old samples due to nonlinear age effects and survivorship effects; survivorship effects may be particularly pronounced in cognitively-intact oldest-old individuals. We have previously referred to this effect as “surviving and thriving” [41]. The present analysis examined associations between self-reported exposure history and brainPAD, as well as brainPAD and three cognitive summary scores, in a cross-sectional sample of cognitively-intact oldest-old adults.

Overall, participants showed structural brain features consistent with delayed brain aging: the mean brainageR-derived brainPAD in unimputed data was −7.99 (SD: 5.37) across all participants, and the mean in females was −9.42 (SD: 4.90). Estimates of brainPAD produced by other commonly-used brain age prediction algorithms were even more negative, likewise consistent with substantially-delayed physiological aging: the mean pyment-derived brainPAD in unimputed data was −14.31 (SD: 4.92). This finding parallels results recently reported by Park and colleagues [32], who observed delayed brain aging in a sample of “superagers” (including oldest-old adults). Collectively these results suggest that some cognitively-intact aging adults may be physiologically younger than birth cohort peers, consistent with a “brain maintenance” model of healthy cognitive aging [38, 44].

While we are unable to conclusively identify specific protective factors in our cross-sectional observational sample, delayed brain aging may be related to protective health behavior or cognitive reserve. Our participants were atypically healthy members of their birth cohort. The lifetime prevalence of myocardial infarction, cardiac arrest, diabetes mellitus, alcohol abuse or dependence, and smoking in our sample was low compared to representative population-based US estimates from comparable birth cohorts [72–75]. The mean educational attainment in the sample was 16.29 years (SD: 3.06), consistent with high cognitive reserve and high adult SES; although associations between educational attainment and brainPAD are inconsistent [10, 15, 76], other aspects of SES (e.g., household income) have been linked to brainPAD [77]. Furthermore, our sample was predominantly non-Hispanic White; several reports have identified racial or ethnic disparities in brainPAD [78–80], although it is currently unclear whether these discrepancies reflect *bona fide* racial/ethnic disparities in brain aging [81], racial/ethnic imbalances in model training data, or some combination of both. Overall, our sample of healthy and cognitively-intact oldest-old participants may represent a best-case scenario for brain function in aging.

Within this highly-selected sample of cognitively-intact oldest-old adults, we did not observe compelling evidence for an association between greater history of deleterious exposures and advanced brain aging (more-positive brainPAD). Self-reported pack-year history, current alcohol use, hypertension diagnosis, high cholesterol diagnosis, cumulative lifetime exercise, BMI, and anticholinergic medication use were not significantly associated with brainPAD; confidence intervals in imputed data were compatible with unstandardized effects ranging from a 3-year delay to a 1-year increase in brain aging (categorical predictors) and a 0.30-year delay to a 0.30 year increase in brain aging (continuous predictors). These findings contrast with prior reports of statistically-significant associations between alcohol use [26], cigarette smoking [12, 23, 24], cardiovascular disease [13–15], and lower physical fitness [10–12] and greater brainPAD in midlife and younger-old samples. Several mutually-compatible mechanisms may explain our findings. First, as previously discussed, our sample was comparatively healthy; few individuals in our sample reported (for example) heavy alcohol use, limiting our ability to detect associations between heavy drinking and brainPAD. Second, brainPAD derived from T1-weighted MR images (as in all 3 algorithms used in the present study) may not fully capture the effects of cardiovascular risk factors on white matter hyperintensity volumes, subclinical infarcts, and microbleeds; replication using multimodal brainPAD would be informative [82]. However, another possible mechanism is suggested by the epidemiology of aging.

Risk factors such as diabetes, hypertension, and obesity show attenuated associations with cognitive status and mortality in oldest-old samples [47–49, 83–85] and in some samples even appear protective [83, 86]; this attenuation has been attributed to survivorship effects. As a birth cohort ages, the least-healthy individuals are disproportionately lost from the cognitively-intact population due to earlier death or incident cognitive impairment. Consequently, surviving oldest-old adults are heavily selected both for overall health and for low susceptibility to common risk factors (e.g., smoking or hypertension): for instance, surviving smokers may be particularly resistant to the adverse physiological impact of smoking. Therefore, exposures harmful on the population level may appear neutral or even beneficial in the oldest-old due to disproportionate attrition of susceptible individuals prior to sampling [42, 87, 88]. This effect, although apparent even in population-based samples of the oldest-old, may be particularly pronounced in cognitively-intact oldest-old adults with high-quality MR data (i.e., the individuals represented in imaging studies of healthy aging).

Notably, female sex was significantly associated with more-negative brainageR-derived brainPAD (i.e., a younger-looking brain). Sex differences in brainPAD have previously been reported; however, the direction of these effects is inconsistent, with some studies identifying more-positive brainPAD in females [36, 89], others identifying more-positive brainPAD or faster longitudinal increase in brainPAD in males [15, 18, 28, 90], and others finding no sex difference [12]. Discrepant results may be partially explained by sex differences in risk factors for brain aging [17, 91] or by systematic differences between brain age algorithms (consistent with the lack of evidence for associations between sex and brainPAD_xgBoost_ or brainPAD_pyment_ in our sample). However, in oldest-old adults, sex-related differences may also be explained by discrepant survivorship and the “health-survival paradox.” Survivorship effects act differentially on men and women: in high-income societies, women typically have longer life expectancies but shorter disability-free life expectancies [92, 93]. Differential selection by dropout or death has been shown to bias observed associations between sex and age-related cognitive decline in longitudinal aging cohorts, such that surviving men are especially healthy (vs. women at the same age) [94]. Similarly, we have previously reported a cross-sectional positive age-GABA association in oldest-old men (but not women) in this sample, potentially due to more stringent survivorship effects acting on aging males (vs. females) [41]. Overall, our findings suggest that sex is likely relevant to at least some aspects of brain aging, but that the nature of this association may vary by birth cohort.

Our sample was largely homogeneous in racial/ethnic composition and high educational attainment. Consequently, we were unable to directly examine aging-related selection effects across racial/ethnic subgroups or socioeconomic strata. However, differential selection may underlie reports of attenuated or reversed racial/ethnic disparities in mortality and morbidity among the oldest-old [95]: for instance, Black oldest-old adults in the United States may be more highly-selected (relative to Black peers) than White oldest-old adults (relative to White peers), resulting in “mortality crossover” [95]. Similarly, racial/ethnic disparities in cognitive health [96] and brain health indices such as white matter hyperintensity volume [97] may be attenuated in older samples. Regarding educational attainment, although higher educational attainment is generally associated with longevity, this association may be comparatively-weak in older cohorts due to increasing educational attainment among successive US birth cohorts, resulting in differing education-related selection effects by birth cohort [98, 99]. Overall, replication of our analysis in diverse samples would provide valuable insight into the impact of aging-related selection effects on brain and cognitive aging across subgroups [100].

The second aim of our analysis was to evaluate associations between brainageR-derived brainPAD and three summary measures of cognitive function in oldest-old adults. Prior studies have reported associations between more-positive brainPAD and worse cognition in lifespan samples [36, 101, 102], midlife samples [103, 104], and typical aging samples [15, 28, 30–32]. While brainPAD-cognition associations have not always been replicated [36, 105] and causality may be temporally complex (i.e., poor early-life cognition has been associated with more-positive brainPAD in adulthood) [103], brainPAD appears to have predictive value for future cognitive decline in at-risk aging adults [33, 34]. Consequently, brainPAD has been interpreted as a potentially clinically-meaningful biomarker of cognitive aging [106].

We did not observe compelling evidence for associations between brainPAD and global cognitive function, coefficient of variation, or crystallized-fluid cognitive discrepancy. This finding may be explained by several mutually-compatible mechanisms. First, the range of cognitive function within our sample was constrained by study inclusion criteria, which may have limited our ability to detect subtle associations across the range of cognitive function in aging. Second, subtle domain-specific associations may not be apparent in global summary measures of cognition, although CoV is typically sensitive to subtle cognitive changes in normal aging and neurological disease [54, 107–109]. Third, although the mean brainPAD corresponded to a roughly eight-year delay in brain aging in the sample overall (using the brainageR pipeline), brainPAD in unimputed data ranged from −24.50 to 6.03. This broad range suggests that our sample may have included both participants resistant to age-related brain changes (i.e., effective brain maintenance) and participants with strong compensatory abilities in the presence of age-related brain changes (i.e., strong cognitive reserve) [4]; that is, several distinct brain aging phenotypes may be represented among cognitively-intact oldest-old adults [110], reflecting potentially dissociable profiles of risk and protective factors [111]. The association between frank neuropathological changes and cognitive function appears to be attenuated in the oldest-old [112–114], consistent with strong compensatory ability in a substantial subset of non-demented oldest-old adults. Our findings, although not definitive, suggest that cognitively-intact oldest-old adults may be a physiologically-heterogeneous group whose cognitive performance may be driven by varying combinations of brain maintenance and cognitive reserve. Follow-up work in larger samples stratified by brain age or educational attainment would contribute to disentangling maintenance vs. reserve pathways in the cognitively-intact oldest-old.

Our cross-sectional results emphasize the need for prospective cohort studies of brain aging in two major ways. First, attrition due to nonrandom mortality and incident cognitive impairment can be directly observed in prospective cohort studies and the resulting biases addressed analytically (e.g., with inverse probability weighting) [115]. Second, it is currently unclear to what extent brainPAD validly predicts future cognitive performance in oldest-old adults. The absence of cross-sectional brainPAD-cognition associations in our sample suggests that biomarkers of physiological aging may be comparatively less associated with cognition in oldest-old age (vs. younger old age) or that heterogeneity in this population may be greater. Balasubramanian and colleagues have previously reported that frank neuropathology is not associated with nonlinear cognitive trajectory over time in oldest-old adults, potentially due to selection for strong compensatory abilities [113]. Our findings raise the possibility that brainPAD may have limited predictive power in this population; longitudinal studies of oldest-old adults with baseline brainPAD observation will be necessary to evaluate its clinical relevance.

Strengths of our study include the use of multiple brain age pipelines to validate findings, as well as use of multiple imputation methods to partially address bias due to missingness of outcome and covariate data (e.g., due to poor MR data quality). However, several limitations should be kept in mind when interpreting our findings. First, lifetime exposures were self-reported retroactively. Consequently, participants may have been misclassified due to inaccurate recall or due to social desirability bias. Additionally, historical information on BMI, alcohol use, and medication exposure in midlife and younger old age was not available; consequently, reverse causality (e.g., weight loss or cessation of alcohol use due to declining health) may have obscured underlying associations between exposures in midlife and brainPAD. Replication with exposure measurements across the lifespan (e.g., from health record data) would be beneficial in characterizing associations between health history and brainPAD; in large samples with well-characterized longitudinal exposure data, latent class trajectory modeling would permit more-detailed examination of exposure trajectories [116]. Furthermore, although we replicated broadly similar patterns of associations using three brain age prediction algorithms, including algorithms with demonstrated predictive value in other samples [34], all three algorithms were T1-based; multimodal imaging models may better capture some aspects of physiological aging [82]. Finally, our relatively-small sample size limited our estimate precision and may have limited our ability to detect weak but real associations; our results should be replicated in larger and more-representative samples of oldest-old adults.

In conclusion, we observed a roughly 8-year delay in brain aging in a sample of cognitively-intact oldest-old adults. Within our sample, brainPAD was not associated with risk factors with well-established relationships to physiological aging at a population level, consistent with atypical resilience among individuals who have survived to oldest-old age. Our findings highlight the potential impact of population- and study-level selection effects on observed associations in aging research. From a translational perspective, our study emphasizes that more research is needed to identify factors that contribute to the survival and resilience of cognitively-intact oldest-old individuals, as these factors may shed light on personalized approaches to the deceleration of brain aging.

## Supplementary Information

Below is the link to the electronic supplementary material.

ESM 1 (DOCX 394 KB)

## Data Availability

All code used in this analysis is available from the Open Science Framework at https://osf.io/ax93d. Data are available from the McKnight Brain Research Foundation upon reasonable request.
