# Supplementary material for: Your brain doesn’t look a day past 70! Cross-sectional associations with brain-predicted age in the cognitively-intact oldest-old
Source: GeroScience. 2025 Dec 11;48(5):7065–82. doi: 10.1007/s11357-025-02027-4 (PMC13601442; doi:10.1007/s11357-025-02027-4)
Supplement: Supplementary file 1 [file 11357_2025_2027_MOESM1_ESM.docx]

TITLE: Your Brain Doesn’t Look a Day Past 70! Cross-Sectional Associations with Brain-Predicted Age in the Cognitively-Intact Oldest-Old

JOURNAL: GeroScience

Mark K. **Britton**, Hannah **Hoogerwoerd**, Joshua **Juhasz**, Keyanni Joy **Johnson**, Paul D. **Stewart**, Pradyumna K. **Bharadwaj**, Stacy S. **Merritt**, Cortney J. **Jessup**, Clinton B. **Wright**, David A. **Raichlen**, G. Alex **Hishaw**, Victor A. **Del Bene**, Virginia G. **Wadley**, Theodore P. **Trouard**, Noam **Alperin**, Bonnie E. **Levin**, Tatjana **Rundek**, Kristina M. **Visscher**, Gene E. **Alexander**, Ronald A. **Cohen**, Eric C. **Porges**, Joseph M. **Gullett**

Corresponding Author:
Mark K. Britton
[mark.britton@yale.edu](mailto:mark.britton@yale.edu%20)
Department of Radiology and Biomedical Imaging
Yale University, New Haven, CT

**Supplementary Table 1.** Covariate imbalance between complete and incomplete cases. Standardized mean differences (continuous) or raw differences in proportion (categorical) are bolded at an absolute threshold of ≥0.10.

| **Characteristic** | **Complete**  N = 113^a^ | **Incomplete**  N = 93^a^ | **SMD**^b^ |
| --- | --- | --- | --- |
| Site |  |  |  |
| 1 | 38 (34%) | 20 (22%) | **-0.12** |
| 2 | 29 (26%) | 16 (17%) | -0.08 |
| 3 | 25 (22%) | 24 (26%) | 0.04 |
| 4 | 21 (19%) | 33 (35%) | **0.17** |
| Age | 88.47 (3.23) | 88.40 (3.02) | -0.02 |
| Female | 60 (53%) | 52 (56%) | 0.03 |
| Race |  |  |  |
| Asian | 0 (0%) | 2 (2.2%) | 0.02 |
| Black/African American | 6 (5.3%) | 0 (0%) | -0.05 |
| Other | 1 (0.9%) | 0 (0%) | 0.01 |
| White | 106 (94%) | 91 (98%) | 0.04 |
| Hispanic/Latino | 4 (3.5%) | 2 (2.2%) | -0.01 |
| Years of Education | 16.29 (3.04) | 15.88 (2.91) | **-0.14** |
| MoCA Score | 24.84 (2.63) | 24.67 (2.31) | -0.07 |
| Current Drinks/Week |  |  |  |
| <7 | 43 (38%) | 37 (43%) | 0.04 |
| ≥7 | 30 (27%) | 18 (21%) | -0.06 |
| None | 40 (35%) | 32 (37%) | 0.01 |
| Lifetime Alcohol Abuse | 1 (0.9%) | 1 (1.1%) | <0.01 |
| Smoking Pack-Years |  |  |  |
| Nonsmoker | 60 (53%) | 34 (43%) | **-0.11** |
| High | 23 (20%) | 25 (31%) | **0.11** |
| Low | 30 (27%) | 21 (26%) | <0.01 |
| Lifetime Hypertension Diagnosis | 66 (58%) | 54 (61%) | 0.02 |
| Lifetime High Cholesterol Diagnosis | 56 (50%) | 43 (46%) | -0.03 |
| Lifetime Diabetes Diagnosis | 13 (12%) | 8 (8.6%) | -0.03 |
| Lifetime Myocardial Infarction | 3 (2.7%) | 1 (1.1%) | -0.02 |
| Lifetime Cardiac Arrest | 2 (1.8%) | 1 (1.1%) | 0.01 |
| Body Mass Index | 25.01 (3.29) | 25.53 (4.16) | **0.14** |
| Cumulative Lifetime Exercise Quartile |  |  |  |
| Q1 | 33 (29%) | 7 (15%) | **-0.14** |
| Q2 | 28 (25%) | 12 (26%) | 0.01 |
| Q3 | 29 (26%) | 11 (23%) | -0.02 |
| Q4 | 23 (20%) | 17 (36%) | **0.16** |
| Anticholinergic Cognitive Burden Scale | 0.58 (1.05) | 0.71 (1.13) | **0.11** |
| Brain-Predicted Age (brainageR) | 80.49 (5.42) | 79.98 (4.08) | **-0.11** |
| Brain-Predicted Age (xgBoost) | 67.68 (3.01) | 68.35 (2.65) | **0.24** |
| Brain-Predicted Age (pyment) | 73.98 (4.59) | 74.48 (4.25) | **0.11** |
| ^a^n (%); Mean (SD) | | | |
| ^b^Standardized mean difference (continuous); raw difference in proportions (categorical) | | | |

**Supplementary Table 2.** Percentage of complete cases and reason for missingness (where applicable) for variables of interest.

| **Characteristic** | **N (%)** | **Reason for Missingness** |
| --- | --- | --- |
| Age | 206 (100%) | — |
| Sex | 206 (100%) | — |
| Race | 206 (100%) | — |
| Hispanic/Latino | 206 (100%) | — |
| Years of Education | 206 (100%) | — |
| MoCA Score | 205 (99.5%) | Incomplete MoCA data (N = 1). |
| Smoking Pack-Years | 191 (93%) | One or both of smoking duration (N = 11) or # of cigarettes per day (N = 11) not reported |
| Current Drinks/Week | 200 (97%) | Not self-reported (N = 6) |
| Lifetime Alcohol Abuse | 202 (98%) | Not self-reported (N = 4) |
| Lifetime Diabetes Diagnosis | 206 (100%) | — |
| Lifetime Hypertension Diagnosis | 202 (98%) | Not self-reported (N = 4) |
| Lifetime High Cholesterol Diagnosis | 206 (100%) | — |
| Lifetime Myocardial Infarction | 200 (97%) | Not self-reported (N = 6) |
| Lifetime Cardiac Arrest | 200 (97%) | Not self-reported (N = 6) |
| Body Mass Index | 203 (99%) | Height and weight not collected (N = 3) |
| Exercise Quartile | 160 (78%) | Incomplete data for Lifetime Physical Activity Questionnaire (N = 45); Lifetime Physical Activity Questionnaire not returned (N = 13) |
| Anticholinergic Cognitive Burden Scale | 198 (96%) | Medications not self-reported (N = 8) |
| Brain-Predicted Age (brainageR) | 157 (76%) | MRI not completed (N = 31); structural MRI failed visual quality control (N = 18) |
| Brain-Predicted Age (xgBoost) | 151 (73%) | MRI not completed (N = 31); structural MRI failed visual quality control (N = 18); processing pipeline not completed (N = 6) |
| Brain-Predicted Age (pyment) | 151 (73%) | MRI not completed (N = 31); structural MRI failed visual quality control (N = 18); processing pipeline not completed (N = 6) |
| UDS 3.0 Global z-Score | 202 (98%) | Either or both of Trails B (N = 4) or Craft Story 21 Immediate Recall, paraphrase (N = 1) not completed |
| UDS 3.0 Coefficient of Variation | 202 (98%) | Trails B not completed (N = 4) |
| NIH Toolbox Crystallized-Fluid Discrepancy | 191 (92.7%) | One or more of NIH Toolbox Picture Sequencing (N = 14), Flanker (N = 12), Pattern Comparison (N = 12), Card Sort (N = 11), List Sorting (N = 11), Oral Reading (N = 12), or Picture Vocabulary (N = 12) not completed |

**Supplementary Table 3.** Linear model predicting brainPAD from participant characteristics on unimputed data, using a listwise deletion approach (N = 125).

| **Characteristic** | **B** | **95% CI**^a^ | **p** |
| --- | --- | --- | --- |
| Age | -0.70 | -1.0, -0.40 | <0.001 |
| Female | -3.5 | -5.9, -1.1 | 0.004 |
| Smoking Pack-Years |  |  |  |
| Nonsmoker | — | — |  |
| High | -1.2 | -4.0, 1.5 | 0.4 |
| Low | -1.5 | -3.8, 0.79 | 0.2 |
| Anticholinergic Medication Use | -0.23 | -2.3, 1.9 | 0.8 |
| Cumulative Lifetime Exercise Quartile |  |  |  |
| Q1 | — | — |  |
| Q2 | -1.0 | -3.8, 1.8 | 0.5 |
| Q3 | -1.4 | -4.2, 1.5 | 0.3 |
| Q4 | -0.78 | -3.9, 2.3 | 0.6 |
| Current Drinks/Week |  |  |  |
| <7 | — | — |  |
| ≥7 | -0.60 | -3.1, 1.9 | 0.6 |
| None | -2.2 | -4.5, 0.16 | 0.067 |
| Lifetime Hypertension Diagnosis | 0.59 | -1.5, 2.6 | 0.6 |
| Lifetime High Cholesterol Diagnosis | -1.3 | -3.4, 0.84 | 0.2 |
| Years of Education | 0.07 | -0.26, 0.41 | 0.7 |
| BMI | 0.07 | -0.25, 0.40 | 0.7 |
| ^a^CI = Confidence Interval | | | |

**Supplementary Table 4.** Linear models predicting brainPAD_xgBoost_ and brainPAD_pyment_ from participant characteristics, fully-imputed data (N = 206).

|  | **xgBoost** | | | **pyment** | | |
| --- | --- | --- | --- | --- | --- | --- |
| **Characteristic** | **B** | **95% CI**^a^ | **p** | **B** | **95% CI**^a^ | **p** |
| Age | -0.92 | -1.1, -0.76 | <0.001 | -0.72 | -0.96, -4.7 | <0.001 |
| Sex |  |  |  |  |  |  |
| Male | — | — |  | — | — |  |
| Female | -0.62 | -1.7, 0.48 | 0.3 | -0.68 | -2.4, 1.1 | 0.4 |
| Smoking Pack-Years |  |  |  |  |  |  |
| Nonsmoker | — | — |  | — | — |  |
| High | 0.01 | -1.1, 1.2 | 1.0 | -2.8 | -2.1, 1.6 | 0.8 |
| Low | -0.13 | -1.3, 1.1 | 0.8 | -0.72 | -2.5, 1.1 | 0.4 |
| Anticholinergic Medication Use | -0.39 | -1.5, 0.7 | 0.5 | -1.3 | -2.8, 0.14 | 0.1 |
| Cumulative Lifetime Exercise Quartile |  |  |  |  |  |  |
| Q1 | — | — |  | — | — |  |
| Q2 | 0.19 | -1.1, 1.5 | 0.8 | -1.7 | -3.6, 0.32 | 0.1 |
| Q3 | 1.3 | -0.09, 2.6 | 0.07 | -0.97 | -3.3, 1.4 | 0.4 |
| Q4 | 1.0 | -0.42, 2.4 | 0.2 | -0.40 | -2.6, 1.8 | 0.7 |
| Lifetime Hypertension Diagnosis | 0.29 | -0.72, 1.3 | 0.6 | -0.80 | -0.76, 2.4 | 0.3 |
| Lifetime High Cholesterol Diagnosis | 0.30 | -0.66, 1.3 | 0.5 | -0.13 | -1.7, 1.5 | 0.9 |
| Years of Education | -0.03 | -0.19, 0.13 | 0.7 | -0.03 | -0.26, 2.1 | 0.8 |
| BMI | 0.01 | -0.12, 0.14 | 0.9 | -0.04 | -0.26, 0.18 | 0.7 |
| ^a^CI = Confidence Interval | | | |  |  |  |

**Supplementary Table 5.** Linear model predicting brainageR-derived brainPAD from participant characteristics, including a sex-by-interaction term to assess sex differences in the impact of education on brain aging, fully-imputed data (N = 206)

| **Characteristic** | **B** | **95% CI**^a^ | **p** |
| --- | --- | --- | --- |
| Age | -0.66 | -0.93, -0.40 | <0.001 |
| Female | -2.18 | -11.1, 6.7 | 0.6 |
| Smoking Pack-Years |  |  |  |
| Nonsmoker | — | — |  |
| High | -0.71 | -2.8, 1.4 | 0.5 |
| Low | -0.99 | -3.0, 1.0 | 0.3 |
| Anticholinergic Medication Use | -0.24 | -2.0, 1.5 | 0.8 |
| Cumulative Lifetime Exercise Quartile |  |  |  |
| Q1 | — | — |  |
| Q2 | -0.63 | -2.7, 1.4 | 0.5 |
| Q3 | -0.55 | -2.9, 1.8 | 0.6 |
| Q4 | -0.18 | -2.5, 2.1 | 0.9 |
| Current Drinks/Week |  |  |  |
| <7 | — | — |  |
| ≥7 | -0.40 | -2.5, 1.7 | 0.7 |
| None | -1.3 | -3.1, 0.51 | 0.2 |
| Lifetime Hypertension Diagnosis | 0.32 | -1.4, 2.0 | 0.7 |
| Lifetime High Cholesterol Diagnosis | -0.52 | -2.2, 1.2 | 0.5 |
| Years of Education | 0.04 | -0.37, 0.45 | 0.8 |
| BMI | 0.05 | -0.18, 0.28 | 0.7 |
| Years of Education x Female | 0.04 | -0.58,0.50 | 0.9 |
| ^a^CI = Confidence Interval | | | |

**Supplementary Table 6.** Fixed effects from linear mixed effects models of brainPAD and cognitive performance, unimputed data.

|  | **UDS 3.0 Global z-score^a^ (N = 156)** | | | **UDS 3.0 Coefficient of Variation^b^ (N = 156)** | | | **NIH Toolbox Crystallized-Fluid Discrepancy^c^ (N = 152)** | | |
| --- | --- | --- | --- | --- | --- | --- | --- | --- | --- |
| **Characteristic** | **B** | **95% CI**^d^ | **p** | **B** | **95% CI**^d^ | **p** | **B** | **95% CI**^d^ | **p** |
| Age | -0.01 | -0.04, 0.01 | 0.4 | 0.00 | -0.06, 0.06 | >0.9 | 0.04 | 0.00, 0.08 | 0.041 |
| brainPAD | 0.00 | -0.02, 0.01 | 0.7 | 0.02 | -0.01, 0.05 | 0.3 | 0.00 | -0.02, 0.02 | 0.8 |
| Years of Education | — | — | — | — | — | — | 0.05 | 0.01, 0.08 | 0.012 |
| Female | — | — | — | — | — | — | 0.03 | -0.20, 0.25 | 0.8 |
| ^a^Adjusted for age, years of education, and sex using standard UDS 3.0 norms ^b^Adjusted for age, years of education, sex, and race/ethnicity using published norms for this composite  ^c^Uncorrected scores were used due to unavailability of age-corrected NIH Toolbox norms for individuals over 85 ^d^CI = Confidence Interval | | | | | | | | | |

**Supplementary Table 7.** Fixed effects from linear mixed effects model of brainPAD and CoV (N = 206), imputed data; individual measure scores were normed for age, years of education, and sex, but demographic adjustments were not applied to the final CoV composite score

|  | **UDS 3.0 Coefficient of Variation^a^** | | |
| --- | --- | --- | --- |
| **Characteristic** | **B** | **95% CI**^b^ | **p** |
| Age | -0.03 | -0.08, 0.02 | 0.18 |
| brainPAD | 0.01 | -0.02, 0.04 | 0.5 |
| ^a^Individual measure scores were adjusted for age, years of education, and sex ^b^CI = Confidence Interval | | | |

**Supplementary Figure 1.** Visualization of xgBoost- and pyment-derived brainPAD and chronological age in cognitively-intact oldest-old adults, unimputed data (N = 151). All brainPAD values were negative, corresponding to delayed brain aging.

Supplementary Methods.

1. *Variable Cleaning*

Cigarette pack-years were estimated from duration of smoking in years and number of cigarettes smoked per day (<1/1/2-5/6-15/16-25/26-35/>35); number of cigarettes/day was estimated numerically using the center of each category (0.5/1/3.5/10/20/30/40) and multiplied by the duration of smoking. Pack-years were categorized (nonsmoker/< median/≥ median) due to the approximately log-normal distribution and high proportion of nonsmokers.

Cumulative lifetime physical activity was assessed using an adapted version of the Lifetime Physical Activity questionnaire [1]. Hours per week (0/0.5/1/1.5/2/3/4-6/7-10/≥11) and months per year (0/1-3/4-6/7-9/10-12) of moderate and strenuous activity were reported for 9 time periods (high school/18 years/25 years/35 years/45 years/55 years/65 years/74 years/the past 3 years). The midpoint of each hour and month category was taken. Hours per week were multiplied by 4 to represent hours per month, then by approximate months per year. Moderate activity was multiplied by 4.5 and strenuous activity by 8.5 to represent METs [2, 3], which were summed to reflect cumulative lifetime activity; cumulative lifetime activity was then categorized by quartile, with Q1 representing the least lifetime activity and Q4 the greatest activity.

Participants self-reported all current medications. Current anticholinergic burden was assessed with the 2012 updated version of the Anticholinergic Cognitive Burden Scale (ACB) [4, 5]. Due to the right-skewness of the distribution, ACB score was binarized for analysis (no anticholinergic medication/anticholinergic medication).

2. *Multiple Imputation*

Multiple imputation of incomplete cases was performed using the MICE algorithm, implemented in mice 3.17.0 [6] and R 4.3.1.

The predictor matrix was developed using the *quickpred* function, which selects predictors for each incomplete variable using Spearman correlations; we selected minpuc = 20% usable cases and mincor = 0.15 minimum rho to achieve a target of 15-25 mean predictors per incomplete variable. The predictor matrix included site, age, gender, and brain-predicted age as predictors for all variables; age and gender were selected due to association with brainPAD in complete-case analyses. After manual editing of the prediction matrix to break feedback loops (e.g., to prevent imputation of binarized lifetime smoking history from reported smoking duration), the mean predictors per incomplete variable was 23.94.

We ran m = 20 imputations and maxit = 20 iterations, using the default burn-in of 5000. The random forest (rf) algorithm was used for all variables. Convergence was determined by visual inspection of model trace plots.

3. Alternate Brain-Predicted Age Pipelines

The pyment brain age prediction algorithm [7] uses the Simple Full Convolutional Neural Network algorithm [8] to predict age as a continuous outcome from raw T1-weighted structural MRI images, which are skull-stripped using Freesurfer’s recon-all pipeline, linearly registered to the MNI152 space in FLIRT, and voxel-intensity normalized before submission to a convolutional neural network; predictions are generated using a linear regression prediction head. The model was trained on a large and diverse sample (53,542 individuals aged 3-95) and its performance evaluated in a test set sampled from reference datasets (N = 10,670) and in an external dataset (N = 2,553). Clinical relevance of the predicted age value was assessed via associations with clinical and biological phenotypes, sociodemographic variables, and lifestyle variables. In our analysis, raw T1-weighted structural MRI images were skull-stripped using FreeSurfer 6.0 and submitted to the pretrained model within a container environment.

The xgBoost brain age prediction algorithm [9] uses linear gradient tree boosting, implemented using the xgboost R package, to predict age from surface-based morphometry and subcortical segmentation variables. T1-weighted structural MR images are submitted to the Freesurfer recon-all pipeline; cortical thickness, area, and volume are generated using the cortical parcellations of the HCP-MMP atlas [10]. Additional subcortical volume variables are derived from the standard Fischl whole-brain segmentation [11]. The algorithm was trained in a large lifespan sample of healthy individuals (N = 35,474; 54% female; age range 3-89) and tuned using fivefold cross-validation to determine the optimal number of training iterations; separate models were trained for males and females. Model quality was assessed by validation on ten independent test samples including patients (N=5,788) and healthy controls (N=4,353). In our analysis, cortical and subcortical variables were extracted using the FreeSurfer 6.0 recon-all pipeline, using the hcp-mmp-b conversion of the HCP-MMP atlas to fsaverage space [12] and the mris_ca_label utility to extract parcellations in subject space. Variables were then submitted to pretrained sex-specific models in R 4.4.1 to generate brain-predicted age estimates.

References

1.	Friedenreich CM, Courneya KS, Bryant HE. The Lifetime Total Physical Activity Questionnaire: development and reliability: Med Amp Sci Sports Amp Exerc. 1998;30(2):266–274. https://doi.org/10.1097/00005768-199802000-00015

2.	Ainsworth BE, et al. 2011 Compendium of Physical Activities: A Second Update of Codes and MET Values. Med Sci Sports Exerc. 2011;43(8):1575–1581. https://doi.org/10.1249/MSS.0b013e31821ece12

3.	Maessen MFH, et al. Lifelong Exercise Patterns and Cardiovascular Health. Mayo Clin Proc. 2016;91(6):745–754. https://doi.org/10.1016/j.mayocp.2016.02.028

4.	Boustani M, Campbell N, Munger S, Maidment I, Fox C. Impact of Anticholinergics on the Aging Brain: A Review and Practical Application. Aging Health. 2008;4(3):311–320. https://doi.org/10.2217/1745509X.4.3.311

5.	Campbell NL, Maidment I, Fox C, Khan B, Boustani M. The 2012 update to the anticholinergic cognitive burden scale. J Am Geriatr Soc. 2013;61(S1):S142–S143. https://doi.org/10.1111/jgs.2013.61.issue-s1

6.	van Buuren S. Flexible Imputation of Missing Data. 2nd ed. Taylor & Francis: New York; 2018.

7.	Leonardsen EH, et al. Deep neural networks learn general and clinically relevant representations of the ageing brain. NeuroImage. 2022;256:119210. https://doi.org/10.1016/j.neuroimage.2022.119210

8.	Peng H, Gong W, Beckmann CF, Vedaldi A, Smith SM. Accurate brain age prediction with lightweight deep neural networks. Med Image Anal. 2021;68:101871. https://doi.org/10.1016/j.media.2020.101871

9.	Kaufmann T, et al. Common brain disorders are associated with heritable patterns of apparent aging of the brain. Nat Neurosci. 2019;22(10):1617–1623. https://doi.org/10.1038/s41593-019-0471-7

10.	Glasser MF, et al. A multi-modal parcellation of human cerebral cortex. Nature. 2016;536(7615):171–178. https://doi.org/10.1038/nature18933

11.	Fischl B, et al. Whole Brain Segmentation. Neuron. 2002;33(3):341–355. https://doi.org/10.1016/S0896-6273(02)00569-X

12.	Faskowitz J. faskowit/multiAtlasTT v0.0.1 v0.0.1 [software]. 2021. https://zenodo.org/record/4459736. Accessed 27 August 2025
